# Management of Fractures in the Edentulous Mandible

**Published:** 2017-06-29

**Authors:** Suraj Jaisinghani, Nicholas S. Adams, Reynaldo D. Rivera

**Affiliations:** ^a^Rochester General Hospital Interventional Radiology Residency, Rochester, NY; ^b^GRMEP/Michigan State University Plastic Surgery Residency, Grand Rapids, Mich; ^c^West Michigan Oral and Maxillofacial Surgery, Grand Haven, Mich

**Keywords:** edentulous, mandible fracture, condyle, body, ORIF

## DESCRIPTION

**Figure 1:**
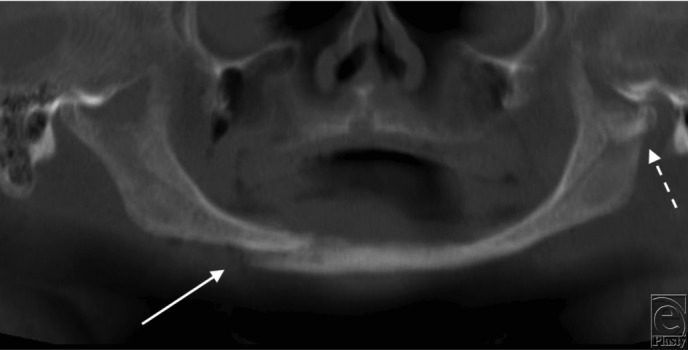
Preoperative panoramic radiographic imaging indicating a right mandibular body (solid arrow) and left subcondylar (dashed arrow) fractures in an edentulous mandible.

**Figure 2. (a, b):**
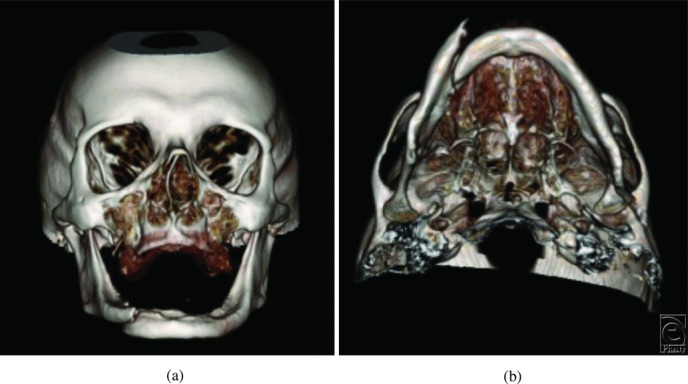
Maxillofacial computed tomography with 3-dimensional reconstruction demonstrating an atrophic, edentulous mandible with fractures of the right body and left subcondylar region.

A 66 year-old man presented to a level 1 trauma center following a motor vehicle rollover. He had loss of consciousness and complained of pain in his lower jaw. After the initial trauma resuscitation, panoramic radiograph imaging (Fig 1) and maxillofacial computed tomography were done (Figs 2*a* and 2*b*).

## QUESTIONS

1. **What are the basic anatomic features of the mandible?**
2. **What are the incidence, causes, and risk factors for mandibular fractures?**
3. **What are the most common surgical approaches to the mandible for open reduction and internal fixation (ORIF)?**
4. **How does the management of mandibular fractures differ in edentulous patients?**

## DISCUSSION

**Figure 3:**
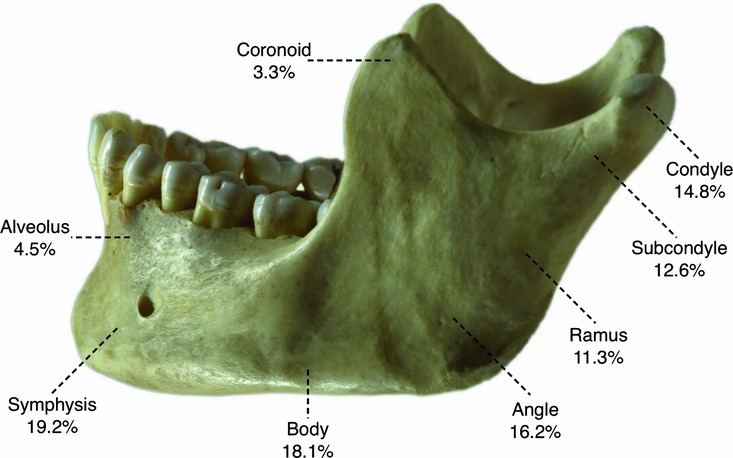
Incidence of mandible fracture by location.^1^ However, in the edentulous mandible, the percentage of body fractures may be higher due to thinner bone in this region. Approximately half of all mandibular fractures are bilateral. Because of the increased instability of bilateral fractures, it is currently recommended that at least one of the fractures be fixed with heavier plates.^2^

The mandible can be divided into several anatomic regions. These include the condyles, coronoid processes, rami, angles, body, symphysis, and alveolus (Fig 3). The coronoid lies anterior to the condyle and is separated by the sigmoid notch. The ramus is located superior to the angle and inferior to the sigmoid notch. The mandibular angle extends to the third molar. The body extends from the mental foramen to the distal portion of the second molar. The symphyseal region is located in the area of the central incisors. The parasymphyseal regions are lateral to the symphysis extending to the mental foramina.

Fractures can be characterized as simple, compound, comminuted, and pathological. Simple fractures include closed linear fractures of the mandible that do not involve tooth-bearing areas. Compound fractures involve tooth-bearing surfaces and are considered open fractures. Comminuted fractures involve fragmentation of the bone into multiple pieces. Pathological fractures are the result of a mandible already weakened by pathologies such as cysts, tumors, or radiation-induced osteonecrosis. Common causes of fractures include assault, motor vehicle accidents, sports-related injuries, falls, and gunshot wounds. Risk factors include age (bimodal), decreased bone density, and systemic pathologies. A large retrospective review found the condylar/subcondylar and parasymphyseal areas to be the most common site for fractures, with the coronoid and alveolar ridges being fractured the least often.^1^ As many as half of all mandibular fractures are bilateral.^2^

Several different incisional approaches can be taken for ORIF depending on the location of the fracture and can be categorized as transfacial or intraoral. Transfacial incision patterns include submandibular, retromandibular, and the preauricular approaches. The submandibular incision allows for exposure to the mandibular body and angle. With this approach, the marginal mandibular branch of the facial nerve is encountered and must be carefully dissected away. A retromandibular incision offers exposure to the posterior border of the ramus and subcondylar fractures. A preauricular incision allows access to the superior portions of the condylar process, including intracapsular fractures.^3^^,^^4^ Intraoral approaches to mandibular fractures are usually made along the lower buccal sulcus. The symphyseal, parasymphyseal, body, angle, and lower ramus can be accessed through this incision. A transfacial trocar or angled drill may be necessary for more distal fractures. Advantages of an open approach over closed treatment include early mobilization, faster healing, and no need for maxillomandibular fixation (MMF). In general, complications associated with ORIF include infection, bleeding, nerve damage, irritation of the tissue overlying hardware, tooth root damage, and malunion.

**Figure 4:**
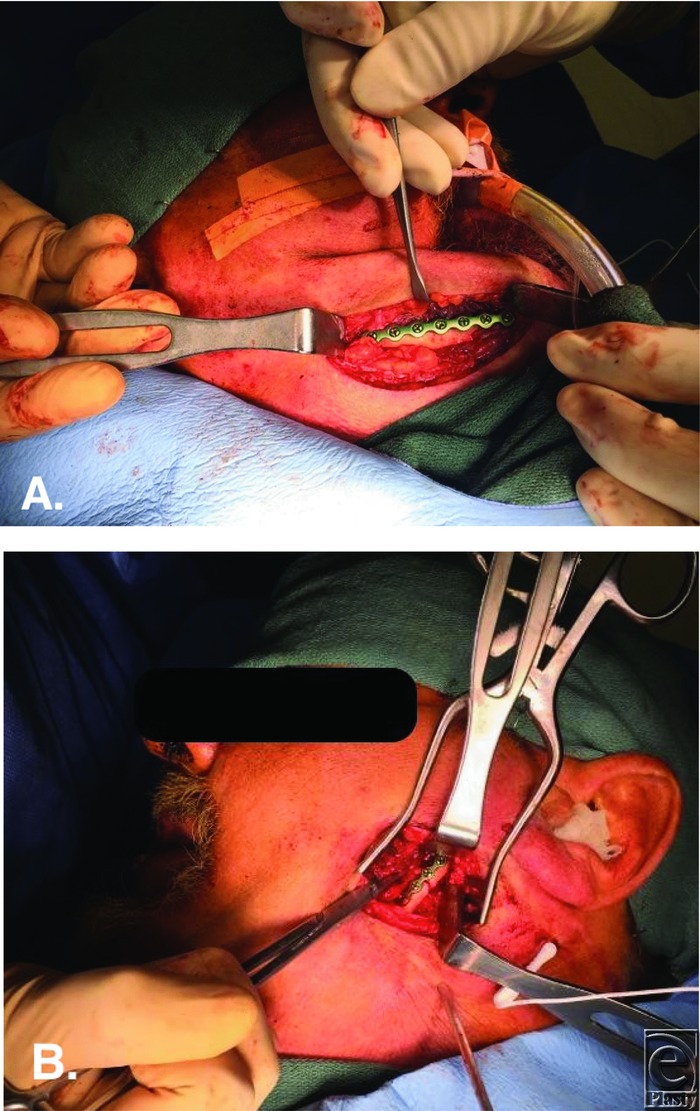
(a) ORIF of the right mandibular body with a locking reconstruction plate via a transfacial, modified Risdon approach. Use of the locking, reconstruction plate provides load-bearing fixation. (b) ORIF of the left subcondylar fracture with a load-sharing miniplate. The fracture was accessed through a retromandibular incision. ORIF indicates open reduction and internal fixation.

Fractures of the edentulous mandible pose unique challenges. The atrophic mandible has little osteogenic potential and a reduced healing capacity (Figs 1, 2*a*, and 2*b*). In the past, MMF was used by wiring the edentulous mandible to dentures or splints. However, because these patients are often elderly with comorbidities, it created additional complications such as infection and pulmonary issues. Transfacial (Figs 4*a* and 4*b*) versus transoral approaches for edentulous mandible fractures differ in their advantages and disadvantages. Currently, there is no consensus on optimal treatment regimens for fractures of the edentulous mandible. Treatment should be individualized to each patient.^5^ Rigid, internal fixation is frequently performed in these patients (Fig 4*a*). This has led to reduced convalescence time and a more manageable healing process. Primary bone grafting is commonly done because of the atrophic nature of the mandible. Options for ORIF include the use of miniplates (Fig 4*b*) or larger locking reconstruction types (Fig 4*a*). Miniplates are small in size, which allows for smaller incision sites. The screws are also small, which allows these plates to be placed in areas of thin bone fragments, such as in the edentulous mandible. Both single and double miniplates can help with load sharing in mandibular fractures. For larger fractures, heavier, load-bearing locking plates can be used. This helps with flexion in the mandible that occurs with opening and closing the mouth, which especially affects the edentulous mandible.
